# Development and internal validation of machine learning models for gouty arthritis classification using routine clinical variables: a retrospective study

**DOI:** 10.3389/fmed.2026.1869807

**Published:** 2026-07-30

**Authors:** Weiwei Ma, Weikang Sun, Zhiyong Hu, Weidong Liang, Huanan Li

**Affiliations:** 1 Jiangxi University of Chinese Medicine, Nanchang, China; 2 Hospital Affiliated to Jiangxi University of Chinese Medicine, Nanchang, China

**Keywords:** clinical prediction, electronic medical records, GA, internal validation, LASSO regression, machine learning, random forest

## Abstract

**Background:**

Gouty arthritis(GA) is a common crystal-induced inflammatory arthropathy influenced by inflammatory, metabolic, renal, hematologic, demographic, and lifestyle-related factors. Because serum uric acid alone may not fully characterize GA in routine clinical settings, integrative models based on routinely available variables may improve disease classification.

**Methods:**

This single-center retrospective study included 7,383 eligible adult participants from the electronic medical record database of the Affiliated Hospital of Jiangxi University of Chinese Medicine between September 1, 2020 and February 1, 2026. GA cases were retrospectively reconstructed according to the 2015 American College of Rheumatology/European League Against Rheumatism classification framework, and non-gout comparators were drawn from a contemporaneous heterogeneous hospital population. Missing predictor values were handled using multiple imputation by chained equations after stratified training/test splitting. Least absolute shrinkage and selection operator regression was used for feature selection. Four supervised learning algorithms were developed and internally evaluated using a 7:3 hold-out design, with cross-validation-based tuning within the training set. Model performance was assessed using discrimination, calibration, decision curve analysis, and confusion matrices.

**Results:**

Among the four models, random forest showed the best overall performance. In the training set, it achieved an area under the receiver operating characteristic curve of 0.8995, accuracy of 0.7062, sensitivity of 0.9623, positive predictive value of 0.6094, and negative predictive value of 0.9419. In the test set, it remained the best-performing model, with an area under the receiver operating characteristic curve of 0.8686, accuracy of 0.6980, sensitivity of 0.9442, positive predictive value of 0.6048, and negative predictive value of 0.9163. Support vector machine ranked second, whereas k-nearest neighbors and logistic regression showed weaker discrimination.

**Conclusions:**

A machine-learning model based on routinely available hospital variables, particularly random forest, showed favorable internal discrimination for GA classification and outperformed conventional logistic regression. These findings support its use as a preliminary screening-oriented classification aid rather than a stand-alone diagnostic tool. External validation remains necessary before broader clinical deployment.

## 1 Introduction

Gouty arthritis (GA) is a common crystal-induced inflammatory arthropathy caused by monosodium urate deposition and is increasingly recognized as a substantial clinical and public health burden because of its recurrent nature, metabolic comorbidities, and impact on quality of life (1, 2). Although hyperuricemia is central to disease pathogenesis, GA in real-world clinical settings cannot be adequately characterized by serum uric acid alone (3). Its occurrence and presentation are shaped by a broader interplay of inflammatory activation, renal handling, metabolic dysfunction, hematologic status, demographic characteristics, and lifestyle-related exposures (4). This multidimensional clinical profile makes accurate identification challenging, particularly in routine hospital practice, where patients often present with overlapping abnormalities rather than a single dominant diagnostic indicator (5). As a result, there is growing interest in approaches that can integrate routinely available clinical information to improve recognition of GA and support more efficient risk stratification.

In this context, machine learning offers a potentially useful solution because it can model complex, nonlinear relationships among multiple variables that may not be fully captured by conventional regression-based approaches (6). However, existing studies on gout-related prediction remain relatively limited, and many rely on variables that are not easily transferable to everyday clinical workflows. To address this gap, the present study conducted a retrospective machine-learning analysis based on a hospital electronic medical record database to develop and internally validate practical classification models for GA using routinely collected demographic, comorbidity, and laboratory variables. A total of 7,383 eligible participants were included. Least absolute shrinkage and selection operator regression was used for feature selection before developing four supervised learning models, including logistic regression, support vector machine, k-nearest neighbors, and random forest. By comparing their discrimination, calibration, and clinical utility, this study aimed to identify a potentially useful internally validated model for GA classification in routine-care settings.

## 2 Materials and methods

### 2.1 Study design and data source

This was a single-center retrospective clinical prediction study based on the electronic medical record database of the Affiliated Hospital of Jiangxi University of Chinese Medicine. The study period extended from September 1, 2020, to February 1, 2026. Demographic characteristics, comorbidities, and routine laboratory data were extracted from the hospital information system. The target outcome was GA status, which was treated as a binary variable. These routinely available variables were used to develop and internally validate machine-learning models for GA classification.

**Figure 1:**
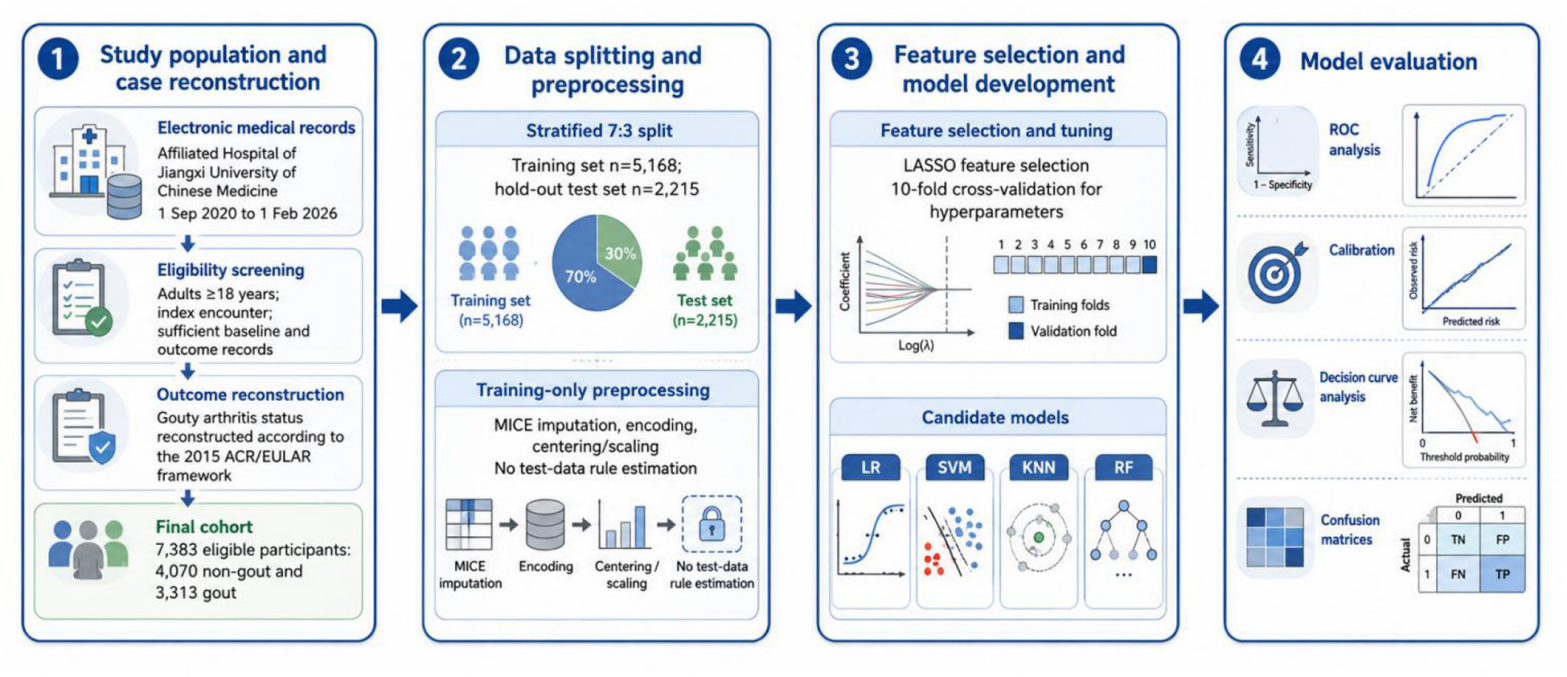
Study workflow and analytic pipeline.

In total, 7,383 eligible participants were included in the final analysis. The overall analytic workflow is summarized in Figure 1. The study was reviewed and approved by the institutional ethics committee of the Affiliated Hospital of Jiangxi University of Chinese Medicine (approval No. JZFYLL20250807082), and the requirement for informed consent was waived because of the retrospective design and the use of de-identified electronic medical record data. The design, analysis, and reporting framework were conducted in accordance with contemporary guidance for clinical prediction modeling and TRIPOD+AI-oriented reporting.

### 2.2 Study population

Participants were screened from the hospital database according to predefined inclusion and exclusion criteria before model development. The inclusion criteria were as follows: (1) age 18 years or older at the index encounter; (2) at least one eligible inpatient or outpatient encounter within the study period from September 1, 2020, to February 1, 2026; (3) availability of sufficient baseline demographic, comorbidity, and routine laboratory information for predictor construction; and (4) sufficient clinical documentation, diagnostic information, laboratory evidence, or imaging-related records to allow retrospective GA label assignment. When multiple eligible records were available for the same participant, only one index record was retained to reduce within-person clustering.

GA cases were retrospectively identified by mapping available EMR information to the 2015 ACR/EULAR gout classification framework (7). The entry condition required documentation of at least one episode of peripheral joint or bursal swelling, pain, or tenderness. When monosodium urate crystals in synovial fluid or a tophus were documented, this evidence was considered sufficient for classification as GA. When crystal documentation was unavailable, available evidence was mapped to the ACR/EULAR domains, including episode pattern, joint distribution, clinical characteristics, serum urate level, and imaging evidence when present. Participants with sufficient evidence to meet the classification threshold were assigned to the GA group. Records with ambiguous or insufficient information for reliable label assignment were excluded rather than forced into either class.

The non-gout comparator group consisted of contemporaneous participants from the same hospital database who did not meet the reconstructed ACR/EULAR GA criteria and had no treating-record evidence strongly supporting GA at the index encounter. This comparator group was intentionally heterogeneous and was not restricted to healthy examinees. It could include routine non-gout clinical encounters, non-gout musculoskeletal complaints, metabolic or general medical visits, and health-examination records, provided that GA criteria were not met and exclusion criteria were absent. Therefore, the classifier was designed to distinguish GA from a real-world non-gout hospital population, rather than from an idealized healthy control population.

The exclusion criteria were as follows: (1) age younger than 18 years; (2) missing or unreliable GA outcome label; (3) records insufficient for retrospective classification; (4) duplicate records or repeat admissions after the index record had been selected; and (5) other joint disorders likely to materially confound gout classification when the treating record clearly favored an alternative inflammatory, infectious, or structural diagnosis, such as rheumatoid arthritis, septic arthritis, psoriatic arthritis, reactive arthritis, calcium pyrophosphate deposition disease, or advanced structural joint disease unrelated to gout. These criteria were applied before training/test splitting and before feature selection to ensure consistent cohort construction and reduce the risk of information leakage.

### 2.3 Candidate predictors and outcome definition

The primary outcome was retrospectively reconstructed GA status. Candidate predictors were selected from routinely available variables in clinical practice to enhance the potential applicability of the model in real-world care. These variables included demographic characteristics (age, sex, marital status, residential location, education, smoking status, and alcohol drinking status), comorbidities (hypertension and diabetes), and laboratory indicators reflecting metabolic, inflammatory, hematologic, and renal status. The laboratory and derived variables included the triglyceride-glucose body mass index (TyG-BMI), white blood cell count (WBC), mean corpuscular volume (MCV), platelet count (PLT), blood urea nitrogen (BUN), glucose, creatinine, total cholesterol (TC), triglycerides (TG), high-density lipoprotein cholesterol (HDL-C), low-density lipoprotein cholesterol (LDL-C), C-reactive protein (CRP), glycated hemoglobin (HbA1c), uric acid (UA), hematocrit (HCT), cystatin C (CysC), and the triglyceride-glucose index (TyG).

TyG was calculated as ln[fasting triglycerides (mg/dL) x fasting glucose (mg/dL)/2], and TyG-BMI was calculated as TyG multiplied by body mass index. TyG-BMI, TyG, glucose, and triglycerides were all retained as candidate inputs because they capture overlapping but not identical representations of metabolic dysfunction. Redundancy among correlated variables was addressed during regularized feature selection rather than by excluding variables *a priori*. Binary clinical variables were recoded as indicator variables for modeling, with sex, higher education, hypertension, diabetes, smoking status, and alcohol drinking status entered as yes/no fields; marital status entered as married vs. divorced/widowed; and residential location entered as urban/town vs. rural/village.

### 2.4 Missing data handling and data preprocessing

Before model development, duplicate records and observations with missing outcome labels were removed. To reduce information leakage, the cleaned dataset was first divided into a training set and a test set by stratified random sampling according to GA status. All subsequent preprocessing operations were derived within the training set before being applied to the held-out test set. Missing predictor values were handled using multiple imputation by chained equations (MICE) under the assumption of missing at random (8). The imputation models were fitted within the training set, and the corresponding imputation rules were then applied to the test set. A prespecified imputation strategy was used: predictive mean matching for continuous variables, logistic regression for binary variables, and multinomial or ordinal regression models for categorical variables as appropriate. Ten imputed datasets were generated with ten iterations per chain. For each imputed dataset, downstream preprocessing was performed within the imputed training-data workflow.

Variable-level predictor missingness ranged from 0.0 to 6.8%; the highest proportions were observed for C-reactive protein (6.8%), glycated hemoglobin (5.6%), and cystatin C (4.3%), whereas all remaining predictors had less than 3.0% missingness. Continuous variables were standardized when required by the modeling algorithm, whereas categorical variables were converted into numeric representations suitable for downstream analysis. Binary variables were coded as 0/1, and ordinal categorical variables were encoded according to their inherent order. The same centering, scaling, and encoding rules learned from the training set were then carried forward to the test set. No preprocessing rule, including imputation, centering, scaling, encoding, or feature-construction procedures, was estimated from the held-out test data, and no outcome-derived feature engineering was performed.

### 2.5 Baseline statistical analysis

Baseline characteristics were compared between participants with and without GA before model development. Continuous variables were summarized as median and interquartile range, and between-group comparisons were performed using the Mann-Whitney U test. Categorical variables were presented as counts and percentages and were compared using the chi-square test. All statistical tests were two-sided, and a *P* value < 0.05 was considered statistically significant. Baseline analyses were descriptive rather than prescreening steps for model inclusion. All statistical analyses were conducted in R (R Foundation for Statistical Computing, Vienna, Austria). The archived workflow used mice for multiple imputation, glmnet for penalized regression, caret as the model-training and resampling interface, e1071/kernlab for support vector machine implementation, randomForest for ensemble-tree fitting, pROC for ROC analysis, rmda for decision-curve analysis, and ggplot2 for figure generation. A prespecified random seed was used for data splitting and training reproducibility.

### 2.6 Feature selection

Feature selection was performed using least absolute shrinkage and selection operator (LASSO) regression in the training set only. LASSO applies penalization to regression coefficients and is commonly used in clinical prediction research to reduce dimensionality, shrink unstable estimates, and retain variables with stronger joint predictive contribution (9). The coefficient profile plot was used to visualize the shrinkage trajectory of candidate predictors as the regularization parameter increased, and 10-fold cross-validation within the training set was used to identify the optimal penalty level. Predictors with non-zero coefficients at the selected penalty value were retained for subsequent model development. This approach allowed the study to reduce redundancy while preserving potentially informative signals distributed across correlated demographic, laboratory, and clinical variables.

### 2.7 Dataset splitting and model development

The full dataset was randomly divided into a training set and a test set at a ratio of 7:3 using stratified random sampling to preserve the distribution of outcome classes between the two subsets. This yielded 5,168 participants in the training set and 2,215 participants in the test set, with broadly preserved case proportions. The training set was used for feature selection, hyperparameter tuning, model fitting, and internal model development, whereas the test set was reserved for hold-out performance assessment.

Four supervised machine-learning algorithms were developed for binary classification: logistic regression (LR), support vector machine (SVM) with a radial basis function kernel, k-nearest neighbors (KNN) using Euclidean distance after centering/scaling, and random forest (RF) based on an ensemble of decision trees. LR was fitted as a standard binomial logistic model using the LASSO-selected predictors and no additional post-LASSO penalization. For SVM, the cost parameter C controlled the penalty assigned to misclassified observations, whereas gamma controlled the width of the radial basis function kernel and therefore the influence range of each observation. For KNN, k denoted the number of nearest neighbors used to assign the class label. For RF, mtry denoted the number of candidate predictors randomly sampled at each split, and ntree denoted the total number of trees in the ensemble.

Hyperparameters were optimized within the training set using 10-fold cross-validation and prespecified grid searches under a fixed random seed (20240201). Specifically, SVM tuning considered cost values from 2^−1^ to 2^5^ and gamma values from 2^−6^ to 2^0^; KNN tuning considered odd values of k from 3 to 21 under Euclidean distance; and RF tuning considered mtry values from 2 to 8 with 500 trees as the base ensemble size (10). The final selected hyperparameters were a standard binomial logistic model without additional post-LASSO penalization for LR, cost = 2 and gamma = 2^−4^ for SVM, k = 21 for KNN, and mtry = 5 with ntree = 500 for RF. Because the outcome prevalence was not extremely imbalanced (44.87% positive), no additional class-weighting or resampling procedure was applied in the primary analysis (11).

### 2.8 Model evaluation

Model performance was evaluated in both the training and test sets, with primary emphasis on the held-out test set. Discrimination was assessed using the area under the receiver operating characteristic curve (AUC), which provided a threshold-independent measure of model performance. Receiver operating characteristic curves were used to visualize discriminatory performance across different thresholds. In addition, threshold-dependent metrics, including accuracy, sensitivity, specificity, positive predictive value (PPV), and negative predictive value (NPV), were calculated at a prespecified predicted-probability threshold of 0.50 to facilitate comparison across models (12). At this threshold, sensitivity was defined as true positives divided by all GA cases, specificity as true negatives divided by all non-gout comparators, PPV as true positives divided by all predicted positives, and NPV as true negatives divided by all predicted negatives. Calibration was assessed graphically using calibration curves to examine agreement between predicted and observed outcome probabilities (13). Decision curve analysis was used to quantify potential clinical utility across a range of threshold probabilities by estimating net benefit. Confusion matrices were constructed to provide an intuitive summary of true-positive, false-negative, true-negative, and false-positive classifications for each model.

## 3 Results

### 3.1 Baseline characteristics of the study population

A total of 7,383 participants were included in the study, comprising 4,070 (55.13%) non-gout comparators and 3,313 (44.87%) participants with GA. Baseline comparisons showed significant between-group differences in several hematologic indicators, including mean corpuscular volume (MCV), platelet count, hematocrit, and hemoglobin; renal, inflammatory, and lipid-related indicators, including creatinine, cystatin C, C-reactive protein (CRP), high-density lipoprotein cholesterol (HDL-C), and uric acid; and demographic, comorbidity, and lifestyle-related variables, including sex, marital status, residential location, hypertension, and smoking status (all *P* < 0.05). By contrast, TyG-BMI, white blood cell count, blood urea nitrogen, glucose, total cholesterol, triglycerides, low-density lipoprotein cholesterol (LDL-C), glycated hemoglobin, TyG, age, diabetes, education level, and alcohol drinking status did not differ significantly between groups (all *P* > 0.05).

Among continuous variables, compared with non-gout comparators, participants with GA had lower MCV, platelet count, creatinine, hematocrit, and hemoglobin, whereas HDL-C, CRP, and cystatin C were numerically higher. Significant between-group differences were also observed for sex, marital status, residential location, hypertension, and smoking status. Uric acid showed only a small between-group difference despite statistical significance, a pattern that may reflect treatment exposure, sampling timing, and the heterogeneous clinical context of routine-care records rather than the absence of biologic relevance. The detailed baseline characteristics are presented in Table 1.

**Table 1:** Baseline characteristics of the study population according to GA status.

Variable	Total (*n* = 7,383)	without GA (*n* = 4,070)	with GA (*n* = 3,313)	Statistic	*P* value
TyG-BMI index, Median (Q1–Q3)	205.34 (179.39, 235.16)	205.67 (179.43, 233.92)	204.97 (179.31, 236.29)	*Z* = −0.49	0.626
White blood cell count (10^∧^3/μL), Median (Q1–Q3)	5.70 (4.79, 6.90)	5.77 (4.80, 6.90)	5.70 (4.70, 6.92)	*Z* = −1.79	0.073
Mean corpuscular volume (fL), Median (Q1–Q3)	92.10 (88.20, 96.00)	92.25 (88.50, 96.10)	92.00 (88.00, 95.80)	*Z* = −2.37	0.018
Platelet count (10^∧^3/μL), Median (Q1–Q3)	200.00 (158.00, 242.00)	203.00 (162.00, 243.00)	197.00 (154.00, 241.00)	*Z* = −3.85	< 0.001
Blood urea nitrogen (mg/dL), Median (Q1–Q3)	15.13 (12.61, 18.21)	15.13 (12.61, 18.21)	14.85 (12.61, 18.21)	*Z* = −0.29	0.772
Glucose (mg/dL), Median (Q1–Q3)	95.50 (88.29, 106.31)	95.50 (88.29, 106.31)	95.50 (88.29, 106.31)	*Z* = −0.87	0.386
Creatinine (mg/dL), Median (Q1–Q3)	0.77 (0.66, 0.90)	0.77 (0.66, 0.91)	0.76 (0.65, 0.89)	*Z* = −2.37	0.018
Total cholesterol (mg/dL), Median (Q1–Q3)	181.85 (159.46, 206.18)	181.08 (158.69, 205.79)	182.63 (160.23, 206.56)	*Z* = −1.78	0.074
Triglycerides (mg/dL), Median (Q1–Q3)	115.04 (83.19, 169.03)	114.16 (82.30, 169.03)	115.04 (83.19, 169.91)	*Z* = −0.41	0.685
HDL-C (mg/dL), Median (Q1–Q3)	50.19 (43.24, 57.92)	49.81 (42.95, 57.14)	50.58 (43.24, 58.30)	*Z* = −2.77	0.006
LDL-C (mg/dL), Median (Q1–Q3)	101.16 (82.63, 119.31)	100.39 (82.24, 118.92)	101.54 (83.01, 119.69)	*Z* = −1.29	0.196
C-reactive protein (mg/L), Median (Q1–Q3)	1.40 (0.80, 2.70)	1.40 (0.80, 2.60)	1.50 (0.80, 2.80)	*Z* = −2.43	0.015
Glycated hemoglobin (%), Median (Q1–Q3)	5.80 (5.50, 6.10)	5.80 (5.50, 6.10)	5.80 (5.50, 6.10)	*Z* = −0.44	0.656
Uric acid (mg/dL), Median (Q1–Q3)	4.80 (4.00, 5.70)	4.80 (4.00, 5.80)	4.80 (3.90, 5.70)	*Z* = −2.10	0.036
Hematocrit (%), Median (Q1–Q3)	41.20 (38.10, 44.60)	41.50 (38.30, 45.00)	40.90 (37.90, 44.10)	*Z* = −5.52	< 0.001
Hemoglobin (g/dL), Median (Q1–Q3)	13.60 (12.50, 14.80)	13.70 (12.60, 14.90)	13.40 (12.40, 14.60)	*Z* = −5.78	< 0.001
Cystatin C (mg/L), Median (Q1–Q3)	0.84 (0.73, 0.96)	0.84 (0.73, 0.96)	0.84 (0.74, 0.97)	*Z* = −2.50	0.013
TyG index, Median (Q1–Q3)	8.63 (8.26, 9.09)	8.63 (8.26, 9.08)	8.62 (8.26, 9.10)	*Z* = −0.13	0.893
Age (years), Median (Q1–Q3)	61.00 (54.00, 68.00)	61.00 (54.00, 68.00)	62.00 (55.00, 68.00)	*Z* = −0.58	0.561
Sex, *n* (%)				χ^2^ = 65.16	< 0.001
Male	3,951 (53.51)	2,006 (49.29)	1,945 (58.71)		
Female	3,432 (46.49)	2,064 (50.71)	1,368 (41.29)		
Marital status, *n* (%)				χ^2^ = 13.84	< 0.001
Divorced or widowed	1,057 (14.32)	527 (12.95)	530 (16.00)		
Married	6,326 (85.68)	3,543 (87.05)	2,783 (84.00)		
Higher education, *n* (%)				χ^2^ = 1.17	0.279
Yes	2,155 (29.19)	1,209 (29.71)	946 (28.55)		
No	5,228 (70.81)	2,861 (70.29)	2,367 (71.45)		
Residential location, *n* (%)				χ^2^ = 13.02	< 0.001
Rural/Village	2,679 (36.29)	1,551 (38.11)	1,128 (34.05)		
Urban/Town	4,704 (63.71)	2,519 (61.89)	2,185 (65.95)		
Hypertension, *n* (%)				χ^2^ = 26.88	< 0.001
Yes	4,782 (64.77)	2,742 (67.37)	2,040 (61.58)		
No	2,601 (35.23)	1,328 (32.63)	1,273 (38.42)		
Diabetes, *n* (%)				χ^2^ = 3.32	0.068
Yes	752 (10.19)	391 (9.61)	361 (10.90)		
No	6,631 (89.81)	3,679 (90.39)	2,952 (89.10)		
Smoking status, *n* (%)				χ^2^ = 18.59	< 0.001
Yes	4,107 (55.63)	2,172 (53.37)	1,935 (58.41)		
No	3,276 (44.37)	1,898 (46.63)	1,378 (41.59)		
Alcohol drinking status, *n* (%)				χ^2^ = 2.65	0.104
Yes	4,002 (54.21)	2,171 (53.34)	1,831 (55.27)		
No	3,381 (45.79)	1,899 (46.66)	1,482 (44.73)		

Continuous variables are presented as median (Q1–Q3) in their original clinical reporting units and were compared using the Mann-Whitney U test. Categorical variables are presented as n (%) and were compared using the chi-square test. TyG-BMI, triglyceride-glucose body mass index; TyG, triglyceride-glucose index; HDL-C, high-density lipoprotein cholesterol; LDL-C, low-density lipoprotein cholesterol; CRP, C-reactive protein; HbA1c, glycated hemoglobin.

### 3.2 Feature selection using LASSO regression

**Figure 2:**
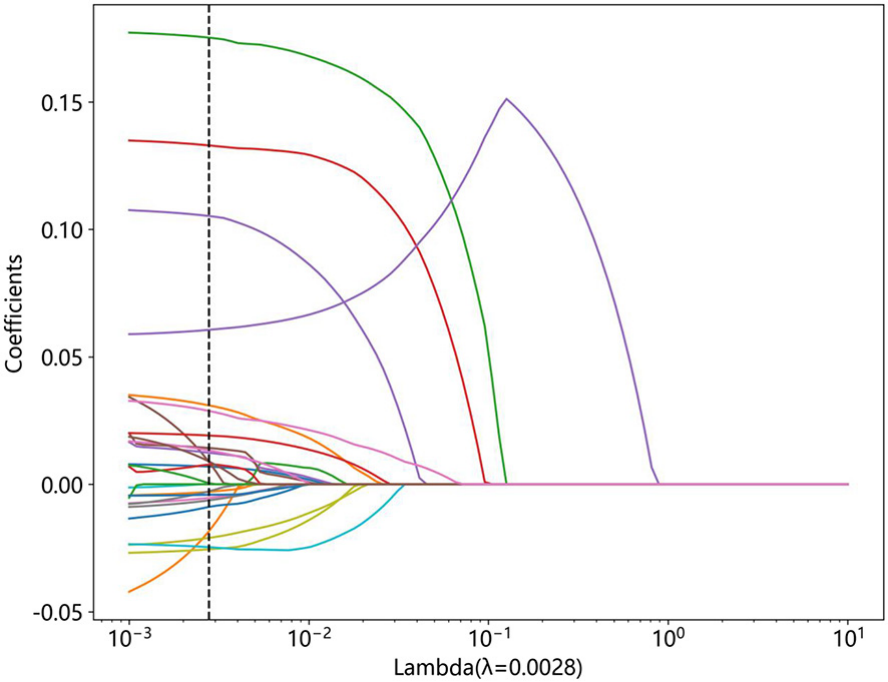
LASSO coefficient profiles of candidate predictors. Each colored line represents the coefficient trajectory of one candidate predictor as the regularization parameter λ changed. The vertical dashed line denotes the selected λ for feature selection. Variables with non-zero coefficients at the selected λ were retained for subsequent model development.

**Figure 3:**
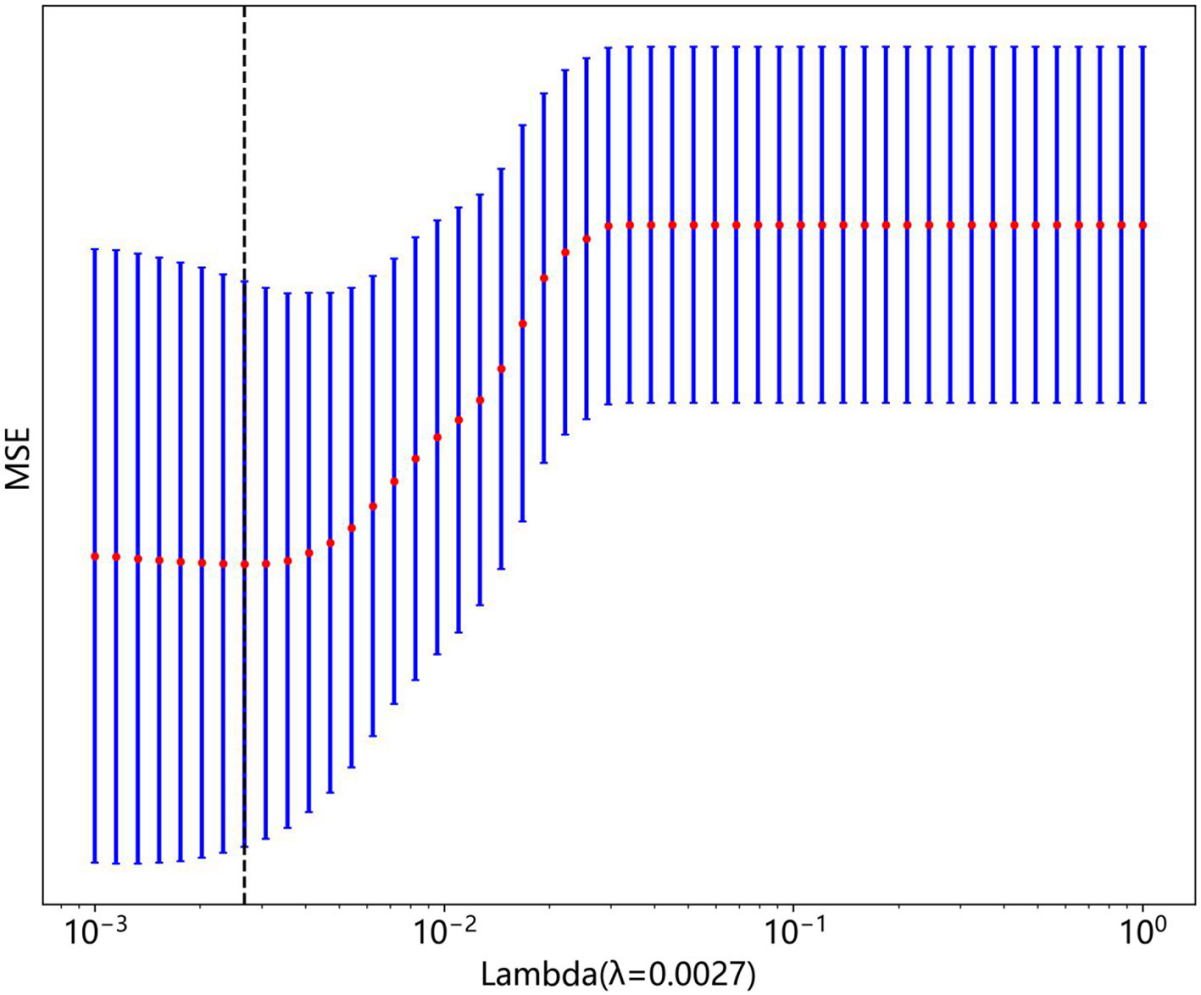
Cross-validation curve for selecting the optimal λ in LASSO regression. The red dots represent the mean cross-validated error at each value of the regularization parameter λ, and the blue vertical bars indicate the corresponding standard error. The vertical dashed line denotes the selected λ value used for feature selection. Variables with non-zero coefficients at this λ were retained as candidate predictors for subsequent model construction.

To identify candidate predictors for model development, LASSO regression was performed in the training set. Feature selection was conducted after the training/test split to minimize selection bias and leakage from the hold-out test set. The coefficient profile plot demonstrated the shrinkage trajectory of candidate variables as the regularization parameter increased (Figure 2). The corresponding tuning process identified the optimal penalty parameter based on the cross-validation error curve obtained within the training data (Figure 3).

**Figure 4:**
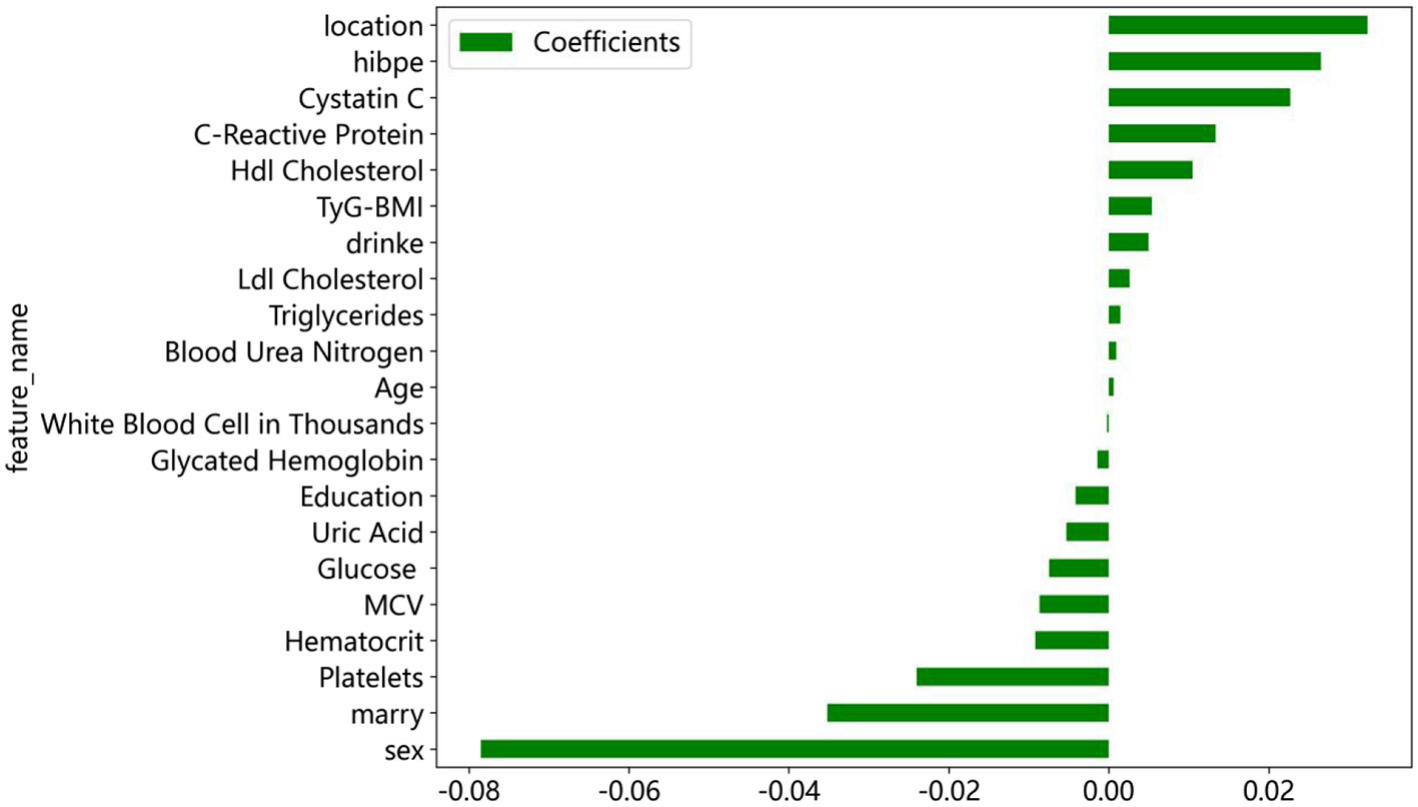
Coefficients of predictors retained by the LASSO model. The bar plot shows the coefficients of predictors with non-zero values at the selected regularization parameter λ. Positive and negative coefficients indicate the direction of association with the outcome according to the coding scheme of each predictor. Variables retained by the LASSO model were considered candidate predictors for subsequent model development.

The feature-weight visualization showed that the retained predictors spanned demographic, clinical, hematological, inflammatory, renal, and metabolic domains (Figure 4). These retained predictors included TyG-BMI, sex, marital status, residential location, hypertension, white blood cell count, mean corpuscular volume, platelet count, blood urea nitrogen, glucose, triglycerides, high-density lipoprotein cholesterol, low-density lipoprotein cholesterol, C-reactive protein, glycated hemoglobin, uric acid, hematocrit, cystatin C, age, education, and alcohol drinking status. This multidomain predictor set was subsequently used for model development and comparison.

### 3.3 Predictive performance of the machine learning models

**Figure 5:**
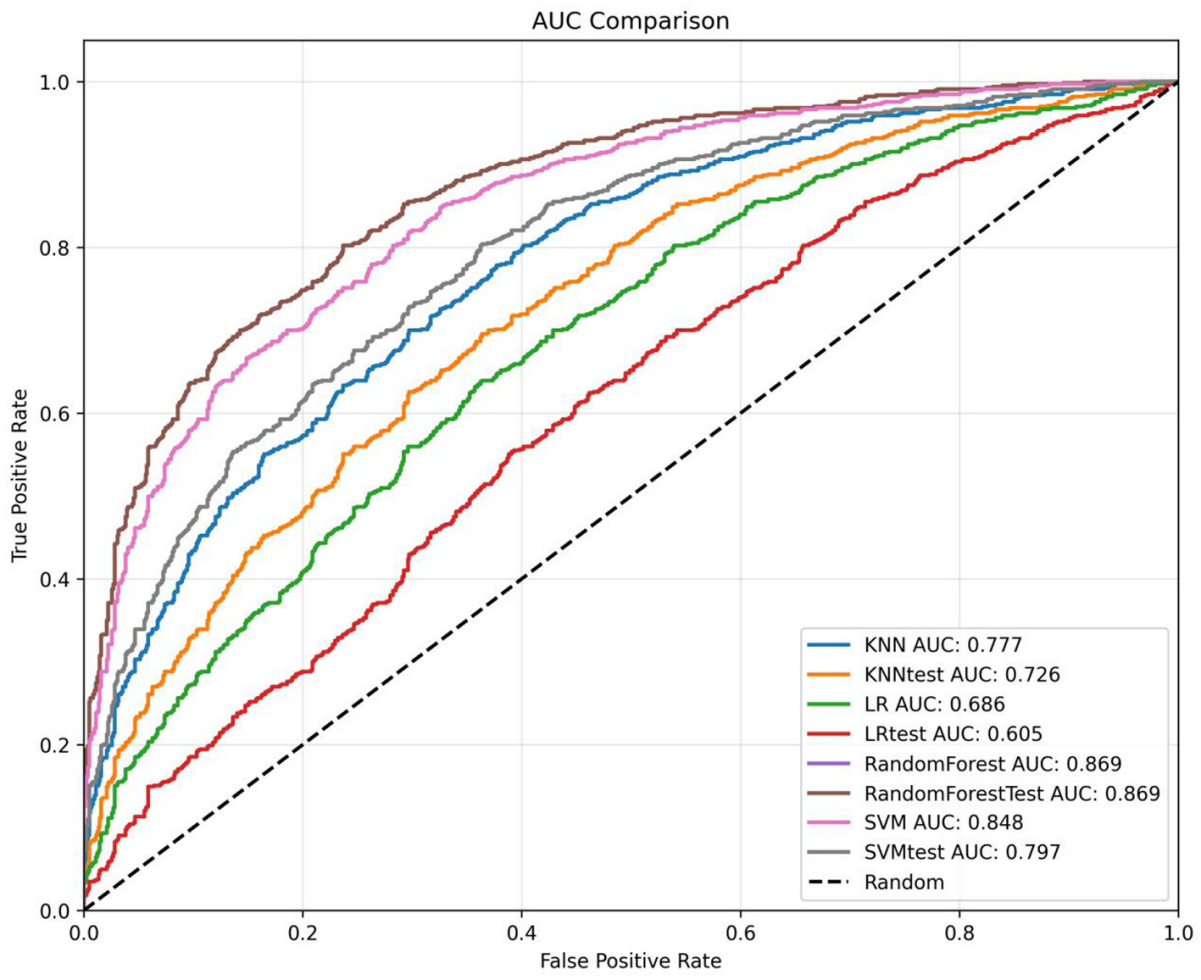
Overall ROC comparison across machine learning models. The random forest model showed the highest overall discriminatory ability, followed by SVM, KNN, and LR.

Four binary classification models were developed using logistic regression (LR), support vector machine (SVM), k-nearest neighbors (KNN), and random forest (RF), and their performance was evaluated in both the training and test sets. Among these models, RF consistently achieved the best overall discrimination performance, followed by SVM, KNN, and LR (Figure 5).

In the training set, RF achieved the highest area under the receiver operating characteristic curve (AUC = 0.8995), with an accuracy of 0.7062, a sensitivity of 0.9623, a positive predictive value (PPV) of 0.6094, and a negative predictive value (NPV) of 0.9419. In the test set, RF remained the best-performing model, yielding an AUC of 0.8686, an accuracy of 0.6980, a sensitivity of 0.9442, a PPV of 0.6048, and an NPV of 0.9163. These results indicated that RF maintained relatively strong discrimination in the hold-out test set, although its threshold-dependent performance should still be interpreted in relation to the fixed probability threshold used for classification.

SVM demonstrated the second-best predictive performance, with AUCs of 0.8483 and 0.7972 in the training and test sets, respectively. KNN showed moderate performance, whereas LR had the weakest discrimination ability, with AUCs of 0.6856 in the training set and 0.6055 in the test set. Accordingly, model ranking should be interpreted primarily from AUC, ROC profiles, decision-curve behavior, and confusion-matrix patterns rather than from a single threshold-dependent index alone.

All threshold-dependent performance metrics in Table 2 were calculated at a fixed predicted-probability threshold of 0.50. Under this threshold, the exported RF metrics corresponded approximately to 2,232 true positives, 87 false negatives, 1,418 true negatives, and 1,431 false positives in the training set, and to 939 true positives, 55 false negatives, 607 true negatives, and 614 false positives in the test set. Given the preserved class counts after stratified splitting, the reported metrics implied very similar true-negative/false-positive counts after rounding across models, which explains the narrow numerical range of specificity. Raw test-set confusion-matrix cell counts (TP, FN, TN, and FP) were 651, 343, 607, and 614 for LR; 882, 112, 607, and 614 for SVM; 805, 189, 608, and 613 for KNN; and 939, 55, 607, and 614 for RF. Therefore, specificity should be interpreted alongside AUC, ROC profiles, decision-curve patterns, and confusion-matrix behavior rather than in isolation.

**Table 2:** Predictive performance of different machine learning models in the training and test sets.

Model	Dataset	Accuracy	AUC	Sensitivity	Specificity	PPV	NPV
LR	Train	0.6114	0.6856	0.7511	0.4977	0.5491	0.7105
LR	Test	0.5680	0.6055	0.6546	0.4975	0.5148	0.6388
SVM	Train	0.6899	0.8483	0.9261	0.4976	0.6002	0.8921
SVM	Test	0.6723	0.7972	0.8869	0.4976	0.5898	0.8438
KNN	Train	0.6628	0.7772	0.8658	0.4976	0.5839	0.8198
KNN	Test	0.6378	0.7263	0.8100	0.4976	0.5677	0.7627
Random forest	Train	0.7062	0.8995	0.9623	0.4977	0.6094	0.9419
Random forest	Test	0.6980	0.8686	0.9442	0.4976	0.6048	0.9163

### 3.4 ROC analysis

**Figure 6:**
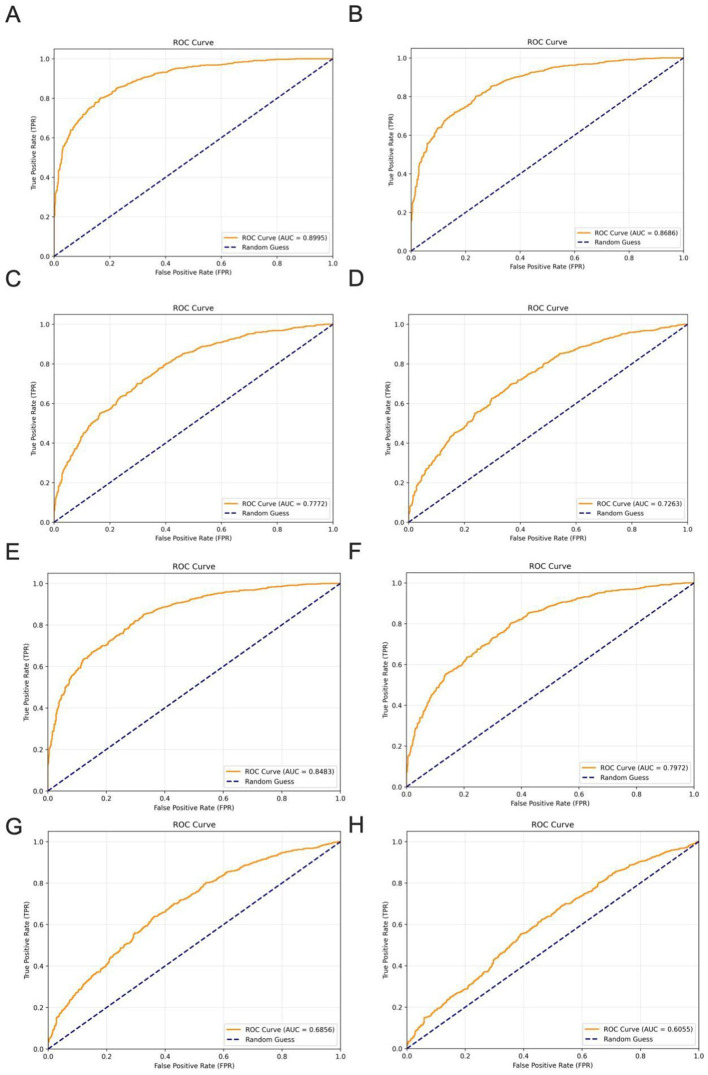
ROC curves of the four machine learning models in the training and test sets. **(A)** Random forest, training set; **(B)** Random forest, test set; **(C)** KNN, training set; **(D)** KNN, test set; **(E)** SVM, training set; **(F)** SVM, test set; **(G)** LR, training set; and **(H)** LR, test set.

Detailed ROC curves for each model in the training and test sets are shown in Figure 6. Random forest demonstrated the most favorable ROC profile in both datasets (Figures 6A, B), whereas SVM showed intermediate-to-good discrimination (Figures 6E, F). KNN exhibited moderate performance (Figures 6C, D), and LR showed the lowest AUC values (Figures 6G, H). Consistent with the quantitative metrics presented in Table 2, these ROC profiles provided visual confirmation of the discrimination ranking across models and supported RF as the strongest classifier in terms of discrimination under the present internal validation setting.

### 3.5 Decision curve analysis

**Figure 7:**
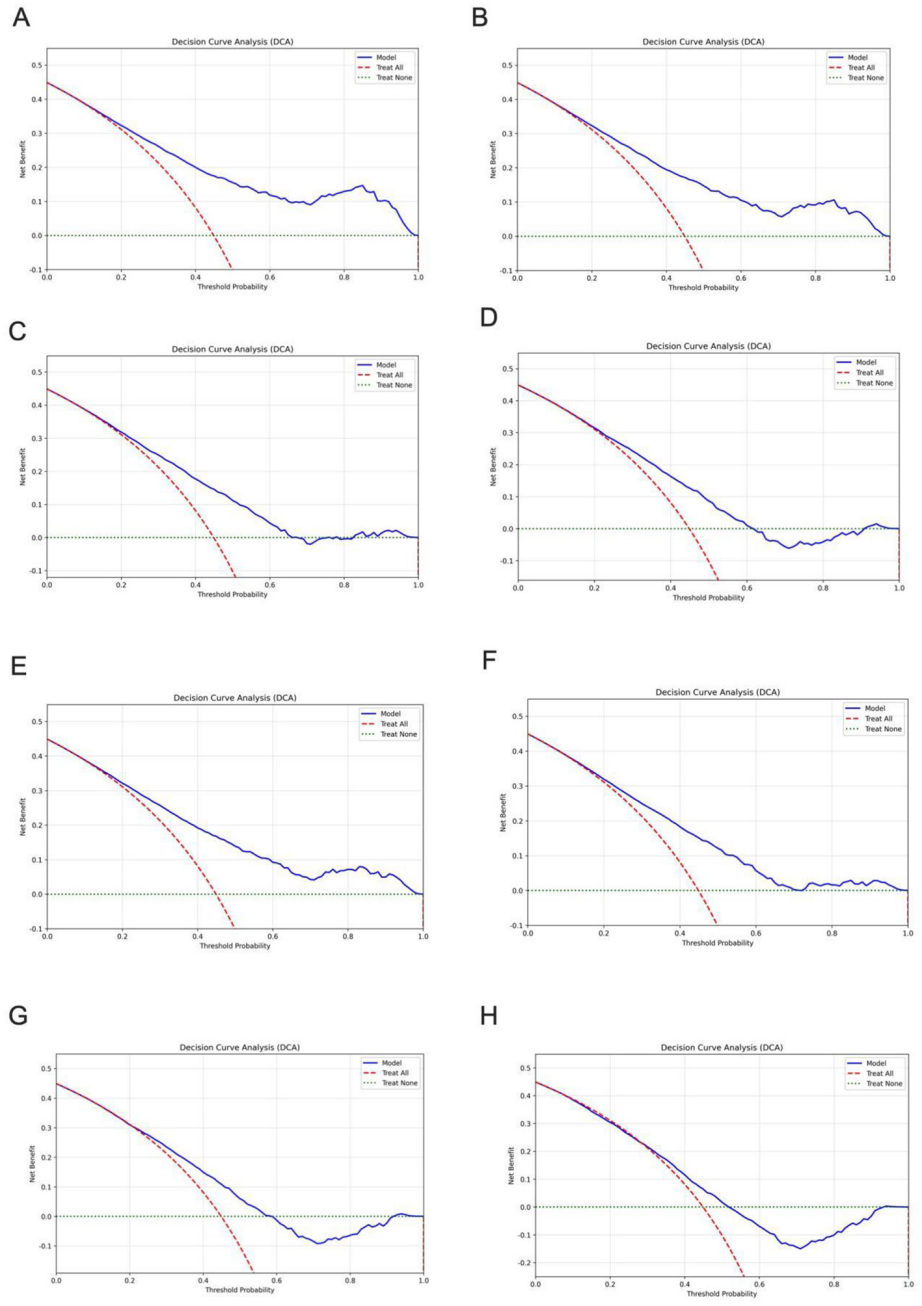
Decision curve analysis of the four machine learning models in the training and test sets. **(A)** Random forest, training set; **(B)** Random forest, test set; **(C)** KNN, training set; **(D)** KNN, test set; **(E)** SVM, training set; **(F)** SVM, test set; **(G)** LR, training set; and **(H)** LR, test set.

Decision curve analysis (DCA) was used to evaluate the potential net clinical benefit of the different models across a range of threshold probabilities (Figure 7). As a complement to discrimination-based metrics, DCA showed that the random forest model had the most favorable net-benefit profile in both the training and test sets, while SVM also demonstrated an acceptable potential utility pattern. The advantage of RF was most apparent across low-to-intermediate threshold ranges, whereas KNN and LR provided relatively smaller net benefit across most threshold intervals. These findings were generally consistent with the ROC-based performance ranking and further supported RF as the most favorable model under the present internal validation setting.

### 3.6 Confusion matrix analysis

**Figure 8:**
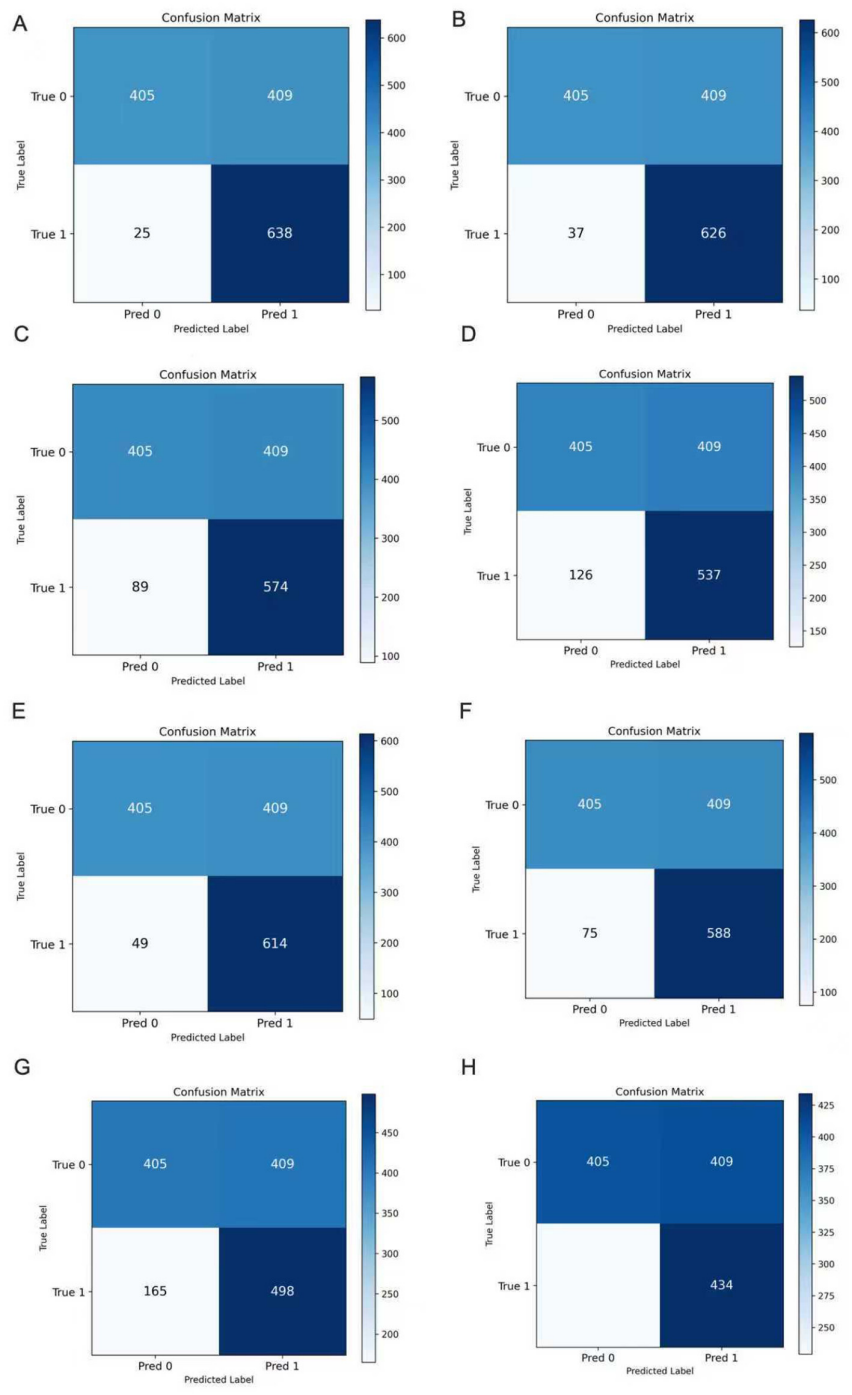
Confusion matrices of the four machine learning models in the training and test sets. **(A)** Random forest, training set; **(B)** Random forest, test set; **(C)** KNN, training set; **(D)** KNN, test set; **(E)** SVM, training set; **(F)** SVM, test set; **(G)** LR, training set; and **(H)** LR, test set.

The confusion matrices of the four models are shown in Figure 8. Random forest and SVM correctly classified a larger number of GA cases as positive than KNN and LR in both the training and test sets, which was consistent with their higher sensitivity values. At the fixed predicted-probability threshold of 0.50, the test-set confusion-matrix counts were TP/FN/TN/FP = 651/343/607/614 for LR, 882/112/607/614 for SVM, 805/189/608/613 for KNN, and 939/55/607/614 for RF. These results indicated that the main threshold-dependent advantage of RF was its higher true-positive count and lower false-negative count, whereas true-negative and false-positive counts were similar across models. In particular, the test-set results for RF (sensitivity 0.9442; NPV 0.9163) suggested that this model generated fewer false-negative classifications than the alternative approaches at the fixed operating threshold.

### 3.7 Calibration performance

**Figure 9:**
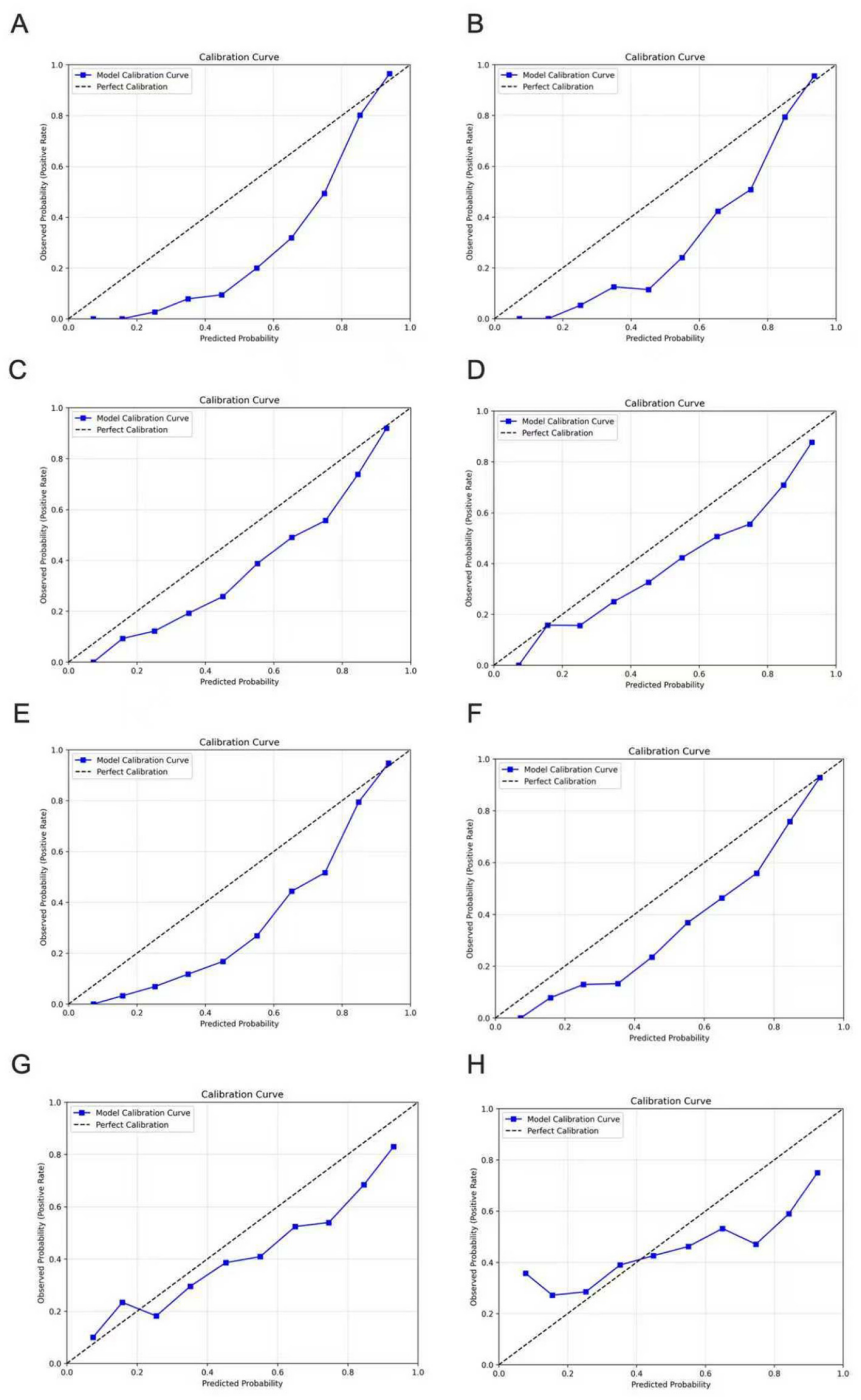
Calibration curves of the four machine learning models in the training and test sets. **(A)** Random forest, training set; **(B)** Random forest, test set; **(C)** KNN, training set; **(D)** KNN, test set; **(E)** SVM, training set; **(F)** SVM, test set; **(G)** LR, training set; and **(H)** LR, test set.

Calibration curves were generated to assess the agreement between predicted and observed probabilities (Figure 9). The graphical calibration performance varied across models, with the random forest model showing relatively closer agreement between predicted probabilities and observed outcomes. SVM and KNN demonstrated moderate graphical calibration with some deviations from ideal agreement, whereas LR showed comparatively weaker calibration consistency.

## 4 Discussion

### 4.1 Principal findings and clinical interpretation

This study aimed to determine whether routinely available hospital variables could be integrated into machine-learning models to improve the classification of GA in real-world clinical settings. The principal finding was that a random forest model provided the strongest internal performance among the four evaluated algorithms, with high discrimination and a sensitivity-oriented classification profile in the held-out test set. This result is clinically relevant because GA often emerges from the convergence of urate deposition, inflammatory activation, renal handling, metabolic status, and host susceptibility, rather than from a single measurable abnormality.

### 4.2 Biological interpretation of the integrated predictor profile

The retained predictors support the concept that GA in routine hospital data reflects an integrated inflammatory-metabolic-renal phenotype. Although hyperuricemia is central to monosodium urate crystal formation, recent mechanistic work has shown that the transition from urate elevation to clinically apparent gout depends on innate immune activation, inflammasome-related pathways, genetic susceptibility, epigenomic regulation, renal urate handling, and cardiometabolic context (14–16). The present findings are consistent with this framework. C-reactive protein captured systemic inflammatory activity, cystatin C and renal-related indices reflected renal functional context, and demographic or lifestyle-related variables such as sex, smoking status, and alcohol drinking status represented host and exposure-level heterogeneity. These variables should not be interpreted as isolated causal determinants in this retrospective model; rather, their combined structure contributed to classification within the hospital population.

The finding that serum uric acid was not the dominant discriminator requires careful interpretation. This result does not weaken the biological role of urate in gout pathogenesis. Instead, it reflects the limitations of a single-time-point urate measurement in a treated, heterogeneous hospital cohort. Serum urate can be affected by urate-lowering therapy, acute flare timing, hydration status, renal function, medication exposure, and the interval between symptom onset and blood sampling. Moreover, many individuals with hyperuricemia do not develop symptomatic gout, indicating that serum urate alone is insufficient to define the clinical phenotype. Metabolomic and genetic studies have similarly suggested that the progression from asymptomatic hyperuricemia to gout involves broader metabolic and environmental influences beyond urate concentration itself (17, 18). Thus, the model's reliance on multiple routine variables is biologically plausible and clinically appropriate.

The retention of triglyceride-glucose body mass index (TyG-BMI), TyG-related metabolic variables, glucose, triglycerides, and lipid parameters further supports the metabolic dimension of GA classification. TyG-related indices are widely used as surrogate markers of insulin resistance and have recently been associated with gout and hyperuricemia in population-based analyses (19). Insulin resistance can influence renal urate excretion, lipid metabolism, endothelial function, and low-grade inflammation, thereby linking metabolic dysfunction to urate-related disease expression. In this study, some metabolic variables showed limited univariate separation between gout and non-gout groups but were still retained in the multivariable modeling process. This pattern is expected in prediction research: variables that appear weak in isolation can improve discrimination when combined with correlated inflammatory, renal, demographic, and lifestyle features. Therefore, the model should be viewed as capturing a multidimensional clinical signal rather than identifying single dominant biomarkers.

### 4.3 Comparison with existing literature and methodological implications

The current findings align with recent advances in gout research showing that gout is a systemic inflammatory-metabolic disease rather than a purely urate-level abnormality. Contemporary reviews have emphasized the interaction among urate biology, innate immune responsiveness, cardiometabolic risk, kidney function, and environmental exposures (20–22). Large genetic studies have identified inflammatory and immunometabolic pathways that extend beyond traditional urate transporter biology (23). Epidemiological studies have also demonstrated that lifestyle exposures, particularly alcohol intake, contribute to gout risk in a sex- and beverage-specific manner (24, 25). The present study complements this literature by showing that routine hospital variables reflecting these domains can be integrated into a clinically useful classification model.

Compared with prior machine-learning studies in gout, this study occupies a distinct methodological and clinical niche. Brikman et al. (26) developed a machine-learning model to predict incident gout among individuals with hyperuricemia using a large nationwide health database and reported useful discrimination with high negative predictive value. That study focused on risk prediction among hyperuricemic individuals. In contrast, the present study addressed GA classification in a heterogeneous hospital population, where the comparator group included non-gout clinical encounters rather than healthy controls alone. This design is closer to real-world hospital triage, where clinicians must distinguish GA from other routine clinical conditions using incomplete and variably timed information. The higher test-set AUC observed in the present study likely reflects differences in study design, outcome prevalence, candidate predictors, and case-control structure; it should not be interpreted as direct superiority over previous models.

The superior performance of random forest over logistic regression also has methodological implications. GA classification is unlikely to follow a strictly linear decision boundary because the disease phenotype arises from interactions among sex, age, renal function, inflammation, metabolic dysfunction, lifestyle exposure, and comorbidity. Random forest can capture nonlinear relationships and higher-order interactions without requiring them to be prespecified. This property likely explains its advantage over logistic regression in this dataset. However, stronger internal performance does not automatically imply clinical readiness. Prediction-model guidance emphasizes that discrimination, calibration, clinical utility, reproducibility, transparency, and external transportability must all be evaluated before implementation (27–30). The inclusion of calibration curves, decision-curve analysis, and confusion matrices strengthened the current evaluation, but external validation remains necessary.

### 4.4 Limitations and future directions

Several limitations should be acknowledged. First, this was a retrospective single-center study based on hospital data, and referral patterns, local clinical practice, and case-mix structure may limit external generalizability. Second, although the use of 2015 ACR/EULAR criteria strengthens phenotype definition, the model underwent only internal hold-out validation based on a single random split; no repeated cross-validation summary, optimism-corrected bootstrap analysis, temporal validation, geographic validation, or fully external validation was performed (31). Third, although missing predictors were handled using a prespecified multiple-imputation strategy and preprocessing was restricted to the training set before transfer to the test set, alternative missing-data assumptions and sensitivity analyses were not exhaustively explored (32). Fourth, several clinically relevant predictors were unavailable or not modeled, including medication exposure, flare stage vs. intercritical phase, disease duration, tophus burden, imaging findings, synovial fluid microscopy, dietary factors, and more granular renal measures (33). Fifth, the operating threshold was fixed at 0.50, which favored high sensitivity but yielded modest specificity; different thresholds may be preferable for alternative clinical use-cases. Sixth, the current manuscript does not provide model-interpretability analyses such as feature-importance ranking or SHAP-based decomposition. Finally, control-group heterogeneity and the retrospective reconstruction of labels from electronic records may also have contributed to residual misclassification.

### 4.5 Summary and outlook

In summary, this study shows that a random forest model based on routinely available hospital variables can provide favorable internal discrimination for GA classification and outperform conventional linear modeling in a heterogeneous hospital dataset. The findings support the use of integrated electronic medical record-based models as preliminary screening aids, while emphasizing the need for external validation, threshold optimization, model explainability, and prospective workflow evaluation before clinical deployment.

## Data Availability

The original contributions presented in the study are included in the article/supplementary material, further inquiries can be directed to the corresponding author.
